# Evaluation of the Implementation and Contribution of Patient Partners on a Steering Committee at a University Hospital in the Province of Québec, Canada

**DOI:** 10.3390/healthcare14132021

**Published:** 2026-07-07

**Authors:** Marie-Pascale Pomey, Seynabou Ka, Monica Iliescu Nelea, Cécile Vialaron, Noé Djawn White, Annabelle Boutin-Wilkins, Marie Chiu-Neveu, Marie-Andrée Côté, Geneviève David

**Affiliations:** 1 Research Centre, University of Montreal Hospital Centre (CRCHUM), Montréal, QC H2W 1R7, Canada; seynabou.ka@umontreal.ca (S.K.); monica.iliescu-nelea.chum@ssss.gouv.qc.ca (M.I.N.); cecile.vialaron.chum@ssss.gouv.qc.ca (C.V.); 2 Health Innovation and Evaluation Hub, Research Centre, University of Montreal Hospital Centre (CRCHUM), Montréal, QC H2X 0C1, Canada; 3 Centre of Excellence on Partnership with Patients and the Public, Montréal, QC H3T 1J4, Canada; genevieve.david.chum@ssss.gouv.qc.ca; 4 Quebec Support Unit for Learning Health Systems, University of Sherbrooke, Longueuil, QC J4K 0B7, Canada; 5 Department of Health Policy, Management and Evaluation, School of Public Health, University of Montréal, Montréal, QC H3T 1J4, Canada; noe.djawn.white.chum@ssss.gouv.qc.ca; 6 Quality, Evaluation, Performance and Ethics Directorate, University of Montreal Hospital Centre, Montréal, QC H2X 0C1, Canada; annabelle.boutin-wilkins.ccsmtl@ssss.gouv.qc.ca (A.B.-W.); marie.chiu.neveu.chum@ssss.gouv.qc.ca (M.C.-N.); marie-andree.cote.chum@ssss.gouv.qc.ca (M.-A.C.); 7 Montreal Campus, École Nationale D’administration Publique, Montréal, QC H2T 3E5, Canada

**Keywords:** patient partnership, patient partners, steering committee, healthcare governance, strategic decision-making, qualitative case study

## Abstract

**Background/Objectives:** Over the past decade, an academic hospital in Montréal has progressively integrated patient partnership into quality improvement committees and peer support. In January 2024, this approach was extended by appointing two patient partners to the Steering Committee, a strategic governance body. This study aimed to describe their integration, examine perceived effects and limitations from patient partners’ and executives’ perspectives, and formulate recommendations for similar initiatives. **Methods:** An in-depth qualitative case study was conducted between August 2024 and April 2025. Semi-structured interviews were carried out with Steering Committee members, including the two patient partners, and with the former Chief Executive Officer. Data were analyzed using thematic content analysis to identify themes related to implementation, participation, perceived contributions, and organizational conditions. **Results:** Integrating patient partners into the Steering Committee was unanimously perceived as relevant and value-adding. Their presence reintroduced the patient perspective, grounded deliberations in lived experience, reinforced the hospital’s mission, supported shared understanding, and encouraged simplification of complex issues. Challenges constrained more active participation, including insufficient clarity regarding roles and objectives; variable access to information due to confidentiality; technical language and acronyms; meeting formats that did not systematically create space for patient partners’ input; and incomplete institutional recognition. Variation across departments also emerged. **Conclusions:** Integrating patient partners into a Steering Committee is a promising governance innovation, but deliberate organizational adjustments are required. Co-constructed expectations and roles, strengthened onboarding and ongoing support, formalized information-access modalities, improved facilitation and plain-language practices, and stronger symbolic and practical recognition are needed to sustain meaningful participation.

## 1. Introduction

Since 2015, the province of Quebec, Canada, has pioneered an innovative approach to patient engagement known as the Healthcare and Services Partnership Model, or the Montreal/Québec Model [1,2,3,4]. At its core, this model recognizes that individuals living with health conditions hold experiential knowledge that is not only distinct from, but complementary to, the scientific expertise of healthcare professionals and managers [5]. Crucially, it proposes that this knowledge be mobilized at every level of a healthcare organization’s governance: strategic, organizational, and operational alike.

At the organizational level, patient and public involvement in governance has gained increasing momentum as a means of making strategic decisions more responsive to patient experience, quality, safety, and accountability. In practice, this involvement may take many forms: participation in patient and family advisory councils, quality improvement and ethics committees, hospital boards of directors, or strategic steering committees. While such arrangements can strengthen the legitimacy and relevance of institutional decisions, empirical studies consistently show that their actual influence depends on a set of conditions, role clarity, access to information, representativeness, decision-making authority, and the willingness of governance bodies to genuinely create space for patient voices [6,7,8,9,10,11,12,13]. Many health organizations are still striving to move patient engagement beyond a merely “token” level [14,15].

Despite this growing momentum, the empirical literature on patient integration at the strategic level, specifically within hospital executive-level Steering Committees, remains strikingly limited. Existing work has largely concentrated on boards of directors [16,17], patient advisory councils [18], or patient and public representation in commissioning and regional authorities [7,10,11,12]. Other studies examine the contribution of patient partners to quality and safety strategic committees [19,20,21] or to research projects [22,23,24,25], while studies of boards of directors tend to focus on how hospitals use patient feedback to formulate strategy and improve care quality [17]. Far less is known about the specific processes by which patient partners are selected, onboarded, supported, and recognized when they join a Steering Committee where decisions involve institutional priorities, resource allocation, confidentiality constraints, and system-level reforms, nor about the organizational factors that facilitate or hinder this integration. This gap matters: without deliberate attention to participation mechanisms and contextual conditions, formal inclusion risks remaining purely symbolic [14,15].

It is within this context that an academic hospital in the heart of Montréal (AHM), a university teaching hospital providing tertiary and quaternary care for the population of Montreal and its surrounding regions, has positioned itself as a provincial leader in patient partnership best practices. The AHM’s Quality Department has developed recognized expertise in identifying, selecting, training, and supporting patient partners (PPs): patients who have received care at the institution and wish to contribute to improving its quality and safety [26]. Traditionally, PPs have been involved in quality improvement committees, departmental head selection processes, new staff onboarding, and certain clinical activities such as peer support [6,19].

Until 2024, however, this involvement had not extended to the Steering Committee itself. In January 2024, the executive leadership took a deliberate step forward by introducing two PPs onto the Steering Committee for one-year renewable terms, under the premise that their participation would strengthen the integration of the patient perspective into the institution’s most strategic decisions. The initiative was led by the Quality Department’s partnership office, a dedicated team responsible for rolling out patient partnership across different levels of governance, in collaboration with the Centre of Excellence on Partnership with Patients and the Public (CEPPP), an organization recognized across Quebec for its expertise in PP engagement at the governance level [27].

This initiative fits within the broader movement toward patient participation in health decision-making. The literature suggests that the presence of patients in governance bodies can surface concerns that would otherwise go unaddressed—around access, continuity of care, communication, and lived experience—while also improving the responsiveness of decisions to real population needs, strengthening their social legitimacy, and increasing the transparency and acceptability of organizational change [1,2,5,6,26].

Building on this foundation, the present article pursues three aims: (1) to analyze the modalities of PP integration onto the Steering Committee; (2) to examine perceptions of the impact of such participation, from both the PPs’ and the steering committee members’ perspectives; and (3) to offer concrete recommendations for the AHM and other institutions seeking to implement a similar organizational innovation.

## 2. Materials and Methods

### 2.1. Theoretical Frameworks

Three complementary conceptual frameworks were used to address patient engagement and the governance of strategic decision-making bodies [28,29,30]. The framework by Pomey et al. [2,3] conceptualizes patient engagement in the health care system at three levels—direct care, organizational design and governance, and policy—and across three degrees of participation: consultation, involvement, and partnership/shared leadership [2,3]. It was specifically used to examine the integration of PPs at the organizational governance level and their influence on the strategic decisions of the committee.

The OECD framework on patient engagement in health system governance [30] complements this approach by describing formal and structured mechanisms through which patients can participate in strategic committees and councils, emphasizing decision-making involvement, legitimacy, and transparency.

Finally, the WHO framework on good governance in health was employed to analyze the broader organizational dimensions of patient integration, including stakeholder participation, transparency, equity, and accountability within decision-making bodies [28,29].

Together, these three frameworks provide a complementary and multi-level lens for examining patient partner integration within strategic governance bodies. While the Montreal Model (Pomey et al., 2015; 2025) [2,3] offers a micro-to-meso level conceptualization of patient engagement, situating PPs along a continuum from consultation to partnership across care, organizational, and policy levels, it does not, in itself, specify the formal mechanisms or governance structures required to operationalize this partnership at the strategic level. The OECD framework [30] addresses this gap by detailing the structural and procedural conditions, such as formal mandates, decision-making rights, and transparent processes, through which patient participation can be embedded into strategic committees and councils. The WHO good governance framework [28,29] further broadens the analysis by situating patient integration within the wider organizational accountability system, encompassing stakeholder participation, equity, and transparency as cross-cutting principles that apply not only to patients but to all governance actors. Used in combination, these three frameworks allow for an analysis that moves from the individual and relational dimension of patient partnership (Pomey et al.) to the formal mechanisms enabling its institutionalization (OECD) and, ultimately, to the broader organizational accountability and governance culture within which this institutionalization takes place (WHO). This integrated approach is particularly suited to studying PP integration into executive committees, as it captures both the depth of engagement achieved and the structural and cultural conditions that support, or constrain, its sustainability.

### 2.2. Research Design

An in-depth qualitative case study [31] was conducted, employing semi-structured interviews with members of the Steering Committee and a focus group that included the two AHM PPs and one PP from another AHM Steering Committee in the province of Québec. The reporting of this qualitative study was guided by the Standards for Reporting Qualitative Research (SRQR) [32]. In addition, all public documents used by the establishment to implement this innovation were collected and analyzed. These documents focused on strategies for integrating PPs into strategic committees.

### 2.3. Participants

All members of the Steering Committee, including all directors (n = 16), the two patient partners, and the Commissioner for Complaints and Quality, were approached to participate in this study. In addition, an invitation was sent to the former president and CEO. The lead author sent an email to all of these individuals to introduce the project and ask if they would agree to participate in an interview. All those approached (n = 20) accepted the invitation, and a date for a Zoom or Teams meeting was scheduled.

Twenty semi-structured interviews were conducted between November 2024 and January 2025 with the sixteen directors, the two PPs, the Commissioner for Complaints and Quality of the AHM, and the former president and CEO, who had made the decision to implement this initiative and had since left the institution.

### 2.4. Data Collection

The interviews were conducted by two researchers (MPP and SK), between November 2024 and January 2025 and focused on the conditions surrounding the PPs’ entry into the Steering Committee, the implementation process, the initiative’s strengths, areas for improvement, perceptions of the impact and potential recommendations. For more details, see Supplementary File S1, which includes the interview guide.

In addition, Steering Committee members were invited to complete a short pre-integration survey to document their prior collaboration with patient partners, the positive aspects and areas for improvement of previous experiences, and any questions, topics, or needs to address before the integration of PPs (Supplementary File S2).

Each interview lasted an average of 30 min and was conducted via videoconference using Zoom or Teams. After obtaining participants’ consent to take part in the interviews, the sessions were recorded and fully transcribed for in-depth analysis. No formal consent forms were required, as the project was conducted as part of a practice evaluation initiative.

### 2.5. Analysis

All interviews were transcribed. The analysis was conducted by two researchers (MPP and SK), and two other co-researchers (MIN and CV) were asked to validate the first level of coding.

The data were analyzed using thematic content analysis [33], guided by a conceptual framework on patient partnership and participation in strategic decision-making bodies. To analyze the data, we followed the six-step guideline by Braun and Clarke [34]. The transcripts were read multiple times to ensure immersion in the data, and initial codes were generated to capture key ideas and patterns related to the integration, roles, and contributions of PPs. Codes were then grouped into broader themes, reflecting both the perceived impacts of PPs on committee discussions and the organizational conditions facilitating or constraining their participation. The analysis was iterative, with regular discussions within the research team to refine themes, resolve discrepancies, and ensure that the interpretations were grounded in the data. QDA Miner Software (version 6.0.2.) [35] was used to manage and organize the coding process.

Upon completion of the coding process, a presentation was given to the five other co-authors to validate the interpretation of the results. Their comments were incorporated into the interpretation. Finally, the results were presented to the Steering Committee to gather feedback, which was incorporated into the findings.

In addition, a thematic analysis of the documents collected was conducted.

This approach allowed for a comprehensive understanding of both the nature and degree of PP participation, as well as the ethical, practical, and organizational factors shaping their influence on strategic decision-making.

## 3. Results

### 3.1. General Results

The directors on the Steering Committee oversee areas including human resources, logistics and infrastructure, nursing, finance, medical and academic affairs, other health professionals, quality and ethics, laboratories, information and telecommunications, public affairs and partnerships, patient access and flow, and teaching. Their tenure ranges from 7 months to over 10 years, providing a wide diversity of perspectives. The PPs have been engaged in partnership activities for approximately six years and have actively participated in Steering Committee meetings since April 2024.

### 3.2. Specific Results

An analysis of the full transcripts and documents revealed three phases that spanned from August 2024 to January 2025: creating a supportive environment; selecting and preparing patient partners; and implementing the intervention (see Table 1). In addition, the findings focus on the perceived effects and limitations of PP participation, as well as the challenges encountered.

A summary of the various preparatory and integration steps is presented in Table 1.

## 4. Creating an Enabling Environment

### 4.1. Conceptualization of the Intervention

The first phase of implementation, from August 2024 to April 2025, focused on conceptualizing the intervention. The Quality Department (QD) and the Centre of Excellence on Partnership with Patients and the Public (CEPPP) [27] reached out to two Quebec institutions with similar initiatives: one had integrated a patient into its Steering Committee, while the other had established a partner-user committee that the Steering Committee could consult. Drawing on these experiences and established guidelines for recruiting and integrating PPs at the governance level proposed by the Ministry of Health and Social Services [36], and the CEPPP guide on identifying and implementing patient partnership mandates [37], the QD and the CEPPP defined the expected role of PPs on the Steering Committee and proposed selection criteria, including motivation, diverse experiences, professional background, and complementarity of skills between the PPs.

Subsequently, a presentation was given by the QD and the CEPPP to Steering Committee members to present the key success factors for such an initiative. No other specific preparation was provided to Steering Committee members to prepare them for welcoming the PPs. Although the members had attended the presentation, some of them did not remember it (n = 4).

*“Prepared? I don’t know. At least, I wasn’t prepared. But did I need to be prepared to welcome them? I consider them full members of the committee.”*

Director

*“I don’t know if we were well prepared. Actually, it doesn’t really require specific preparation, but rather openness. Because we’re used to working in isolation, thinking: ‘Confidential information, we can’t talk about it.’”*

Director

For others (n = 3), there was not enough time to clarify the objectives of the PPs’ participation, their roles, the terms of their involvement in the discussions, and the expectations for both patients and directors.

*“They arrived one day, we were introduced to them, and that was it. Preparation? I missed it, I’m cautious. Because sometimes… In any case, if there was any preparation beyond: ‘We’re going to appoint patient partners, and in the next session, we’ll introduce PP1 and PP2,’ that’s about it. What should be done, I think now it’s done—they’re here—but still, clarifying their role.”*

Director

All the interviewees emphasized the efforts of the Quality Department and Executive Management to raise awareness among directors about the importance of this initiative.

*“It was the CEO who told us he wanted to bring patient partners to the Steering Committee table, with an argument… that, for him, this was what he had done elsewhere in his previous institution.”*

Director

*“Had I heard about it beforehand? I think it was presented by the director of the DQEPE one or two weeks before.”*

Director

In addition, a questionnaire was administered to the directors to assess their previous experience working with patient partners and any concerns they might have regarding the participation of patient partners on the Steering Committee. Eleven directors responded (response rate: 68%), of whom 90% (10/11) had previously collaborated with at least one PP in a project or committee. The results of this questionnaire are presented in Table 2. The survey instrument is provided in Supplementary File S2.

Based on these results, some key facilitators and gaps can be identified (see Box 1).

Box 1 **Facilitators and gaps in creating an enabling environment.**

**Strengths**

**Limitations**
Explicit, visible support from senior management (CEO sponsorship)Limited awareness among committee members of the potential contribution of PPs, persisting despite the awareness session A lack of structured training explaining why PP staff were recruited and the objectives being pursuedHigh prior exposure to PP collaboration by directorsLow recall of the awareness sessionStrong CEO/QD-driven legitimizationInsufficient time to clarify roles & expectations Widely shared sense that “openness” matters more than formal prepNo follow-up or reiteration after the initial sessionDedicated resources (QD + external partnership organization (CEPPP))Perception of insufficient preparation time to welcome the PPs

### 4.2. Selecting and Preparing Patient Partners

A call for applications was sent to all AHM PPs and the members of the patient and family committee, saying that the establishment was recruiting two PPs on the Steering Committee. Of the 14 PPs who applied, 3 candidates were interviewed by a selection committee, composed of the CEO and the quality director who used selection criteria established during the design phase of the initiative. These criteria included: (1) having served on AHM committees in recent years; (2) the ability to express oneself easily in a strategic setting; (3) having been exposed to strategic issues in one’s professional life; (4) a willingness to devote the necessary time to participate in meetings (see Supplementary File S3). As a result, two patients were selected to serve on the Steering Committee, and their characteristics are summarized in Table 3.

In parallel, several integration activities were implemented for the PPs. They received training on how the Steering Committee operates and their role on it, signed a confidentiality agreement, and were provided with an AHM email address giving them access to the Steering Committee Teams channel and related meeting documents:

*“After my candidacy was confirmed, I received several documents describing the structure, committees, and departments.”*

PP

*“There was a lot of goodwill from the Quality Management Department to provide proper guidance. Personally, I think I had a head start due to my involvement since 2019 (…) I really came with a range of experience, so I wasn’t starting from zero.”*

PP

*“We were trained by the quality management team; it would have been nice if patient partners had also been there to train us, and also on the selection committee. But I really appreciated that there were two of us going through all these steps so we could discuss things together.”*

PP

Based on these results, some key facilitators and gaps are summarized in Box 2.

Box 2 **Facilitators and gaps in selecting and preparing patient partners.**

**Strengths**

**Limitations**
The CEO’s presence on the selection committeeNot having PP representatives on the selection committeeRecruitment of two PPsPPs stressed the importance of being trained by other PPs (peer-led training)Complementarity between the two PPs’ profiles
Being able to dialogue with another PP during preparation was valued
Prior experience serving on committees

### 4.3. Implementing the Intervention

#### 4.3.1. PPs Welcome

The PPs began participating in Steering Committee meetings in April 2024. Until September 2024, they received specific support to facilitate their integration.

*“Upon their arrival, we coached them: who’s who, how we operate, the tacit and non-tacit rules of the game.”*

Director

*“During the first few months, we had lunch together after the Steering Committee meetings to translate what was being discussed, including the things left unspoken.”*

Director

Directors (n = 4) also took time to answer PPs’ questions before and after meetings, with these informal moments seen as essential to the quality of their welcome: *“One should not underestimate the exchanges before and after Steering Committee meetings to properly welcome the PPs.”* Director.

#### 4.3.2. Perceptions of Steering Committee Members

Most directors (n = 12) welcomed the initiative, considering it consistent with AHM’s values and ongoing partnership efforts:

*“I think we’re mature enough for this.”*

Director

*“I received it very positively. It’s a great idea; I had never seen this done elsewhere.”*

Director

A few directors (n = 4) initially expressed reservations, mainly related to the novelty of the initiative and practical concerns (PPs’ ability to contribute, access to confidential information, timing relative to leadership transitions):

*“At first, it was a surprise. I wasn’t really sure how to approach them.”*

Director

*“Will they be able to do it? That was the main question.”*

Director

They also raised concerns about overburdening patients with responsibility for healthcare costs: *“It’s good that they are aware, but do we really want them to carry that weight?”* Director.

After integration, all directors adopted an observational and adaptive stance, with no overt resistance; initial concerns dissipated quickly:

*“My concerns evaporated fairly quickly.”*

Director

The process followed by directors from reservation to acceptance is summarized in Box 3.

Box 3 **From Reservation to Acceptance.**

Initial concerns (n = 4) → Observation/adaptation (all directors) → Recognition of added value Underlying facilitator: an organizational culture already sensitized to partnership.

#### 4.3.3. Perceptions of PPs

From the PPs’ perspective, the welcome was perceived as very positive, aided by prior experience in strategic governance settings.

*“We were very well received. The directors are open and attentive.”*

PP

*“I’ve been involved in numerous strategic committees at the university and research levels (…) I knew how they worked.”*

PP

Based on these results, the key facilitators and gaps in the reported onboarding context are summarized in Box 4.

Box 4 **Facilitators and gaps in onboarding and initial integration.**

**Strengths**

**Limitations**
PPs’ own recognition that the directors were open to their arrival.Difficulty understanding vocabulary and acronyms, and accessing documents, limiting PPs’ ability to intervene in discussionsPPs having previously presented to committees to follow the discussions of the Steering Committee.

### 4.4. Perceived Effects

#### 4.4.1. Nature of the Discussions

All directors indicated that the presence of the two PPs did not change the nature of discussions; none reported avoidance or self-censorship: *“No one holds back from saying what they need to say because ‘there are patient partners’ (…)”* Director.

Some directors noted that certain PP interventions were sometimes less relevant when overly academic or outside their expected role: *“Sometimes, some comments stray a bit from the expected role of patient partners (…) They need to wear a specific hat, not just the patient hat.”* Director.

Clarifying questions on vocabulary and acronyms raised by PPs contributed to collective understanding (n = 14): *“PPs feel comfortable asking, ‘What does this mean?’—something directors don’t always do.”* Director. In fact, thanks to the questions asked by the PPs, directors who did not understand certain topics covered can now participate more easily in the discussions.

#### 4.4.2. Reminder of the Patient Perspective

The presence of PPs is unanimously seen as essential (n = 18), acting as guardians of the patient perspective: *“Now that they are present, it forces us to consider the patient dimension.”* Director.

In the context of budget optimization, PP interventions helped reframe issues, leading for instance to the adoption of a patient risk gradation for financial optimization measures: *“Are you actually evaluating the impact of these measures on patients?”* PP.

#### 4.4.3. Nature of the Missions of a University Hospital

Some directors (n = 5) noted that PPs reinforce the core missions of a university hospital, particularly research and innovation, including during periods of financial restraint: *“How is it that the AHM doesn’t have more AI projects? We should be ahead of the curve.”* PP.

#### 4.4.4. Positioning on Key Issues

Directors (n = 6) appreciated PPs’ empathy regarding management challenges and constructive attitude: *“They understand our challenges, and they are neither vindictive nor aggressive.”* Director.

#### 4.4.5. Reflections on the Role of PPs Across Departments

The presence of PPs on the Steering Committee prompted department directors to reflect more broadly on the role of PPs within their own departments. Depending on the department, the relevance of this presence was viewed differently (see Box 5).

Box 5 **Variation in PP Relevance by Department Type.**

**Department Type**

**Perceived Relevance**

**Illustrative Quote**
ClinicalHigh, though few concrete examples yet
*“We would benefit from having someone on the clinical committee to bring that voice.”*
Operational/technicalModerate, topic-dependent
*“In hygiene and sanitation, there would be a gain. For building issues, less so.”*
Support servicesIncreasing, used to bring teams closer to patients
*“Bringing my team closer to the patient would make a big difference.”*
Finance / HRLow but growing interest
*“We hire a lot of people, so it seems like it would be complicated to implement, but we could improve.”*

A gap persists between the strongly positive perception of PPs’ contribution to decision-making and their actual integration into departments.

A summary of the perceived effects is presented in Box 6.

Box 6 **Six Key Observations on PP Participation.**

No change in the nature of discussions (no self-censorship)PPs as a constant reminder of the patient perspectivePPs as a reminder of the university hospital’s mission (research, innovation)PPs’ positioning on managerial issues (empathetic, constructive)PPs improving collective understanding of technical issuesUneven integration of PPs across Department

### 4.5. Challenges Encountered

#### 4.5.1. Clarity of Roles and Objectives

Some directors (n = 4) reported difficulties understanding the precise role expected of PPs: *“The objectives were not clear at all. I kind of made them up myself. They were never clearly stated.”* Director.

From the PPs’ perspective, the absence of an explicit definition of objectives and roles complicates integration: *“Objectives must be clearly defined, both with the patient and with the management team (…).”* PP.

Many participants (n = 9) felt this lack of clarity limits the full potential of PPs’ presence; co-construction involving both management and PPs would help.

#### 4.5.2. Confidentiality

Although PPs sign confidentiality agreements, they face limits in accessing information.

*“There are confidential files with our supervisory bodies that patient partners do not have access to.”*

Director

*“I signed everything. If there is no trust, why are we here? It sends a mixed message.”*

PP

These constraints also had symbolic effects, such as PPs being excluded from internal communications: *“It’s a mailing list oversight, but it gives the impression that they aren’t part of the committee.”* Director.

#### 4.5.3. Participation and Contribution

Directors (n = 8) emphasized the importance of creating an environment that encourages PPs to speak: *“Reserving a short slot in the agenda for PPs would help value their presence and encourage their contributions.”* Director.

#### 4.5.4. Language Used

Patients pointed out that they had difficulty following certain discussions because they did not understand the vocabulary or acronyms used. This situation can hinder understanding and active participation.

*“The healthcare system is an alphabet soup. Even for us, it’s not always easy.”*

Director

*“I still find myself checking acronyms during meetings.”*

PP

#### 4.5.5. Recognition of Their Presence on the Steering Committee

Regarding PPs’ actual status, the ambiguity between invited members and full members creates situations that are less than ideal for their ability to feel included in the Committee: *“We are told we are Steering Committee members, but in reality, it’s not that simple.”* PP.

#### 4.5.6. Healthcare System Reform

Directors (n = 13) believed the new integrated provincial health organization (Santé Québec) does not threaten PPs’ presence on the Steering Committee, though current security and confidentiality rules remain a barrier to full digital integration: *“Integrating patient partners aligns with current directions, but we are waiting to see the official guidelines.”* Director.

Several directors (n = 8) noted that the results of this evaluation could encourage other Steering Committees in the network to integrate PPs: *“If the study shows more benefits than drawbacks, it could support some reflection at Santé Québec.”* Director.

Box 7 summarizes the challenges encountered.

Box 7 **Summary of Challenges.**

**Challenge**

**Core Issue**
Clarity of roles and objectivesNo reiteration after the initial session; ambiguity persistsConfidentialityRestricted document access despite signed agreementsParticipation and contributionPPs’ input not systematically solicitedLanguage usedAcronyms and jargon hinder participationRecognition of presenceSymbolic gaps (e.g., absence from website listing)Healthcare system reformUncertainty linked to Santé Québec’s new security/confidentiality rules

**Figure 1:**
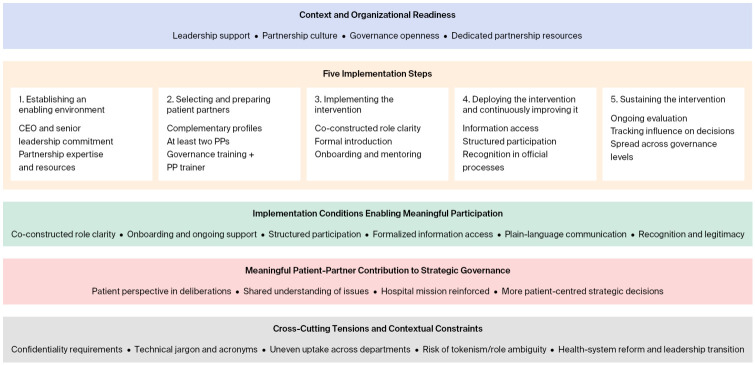
Conceptual implementation model for integrating patient partners into a hospital Steering Committee.

Together, these findings are summarized in the conceptual implementation model presented in Figure 1.

## 5. Discussion

This study aimed to analyze the conditions for the integration and contribution of PPs on the Steering Committee of an academic hospital in Quebec, Canada, based on the cross-perceptions of both the executive members and the PPs themselves. It complements some front-line studies [38] or regional authorities [7] that highlight the contribution of PPs in strategic committees. The results underscore an overall high acceptance of the PPs’ presence and a clear recognition of the value they add, while also revealing organizational and relational tensions influencing the actual scope of their participation.

### 5.1. High Acceptability but Incomplete Integration

Overall, the integration of PPs into the Steering Committee was positively perceived by the majority of directors interviewed. This acceptance aligns with the AHM’s organizational values and previous patient partnership initiatives. PPs are recognized as providing a complementary perspective that can inform strategic decisions through the lens of patient experience.

However, the findings show a gap between the principled support for partnership and its operationalization within governance mechanisms. This tension, widely documented in the literature on patient and public involvement at strategic decision-making levels, highlights the risk of participation being limited to a symbolic or consultative role when the necessary organizational, structural, and relational conditions are not fully met [14,26,39,40,41].

### 5.2. Clarity of Roles and Objectives as a Central Condition for Partnership

A major finding concerns the lack of clarity regarding the role and objectives assigned to PPs within the Steering Committee. Both the directors and the PPs reported the absence of an explicit and shared definition of what was expected from their participation. This situation led some actors to individually reconstruct the meaning and purposes of the initiative, generating heterogeneous expectations.

The literature emphasizes that a co-defining of roles and expectations by PPs and professionals is a key lever for supporting active, legitimate, and sustainable participation, particularly in decision-making spaces, by fostering mutual recognition of expertise and clarity around the expected contributions [8,9,42]. Without this clarification, PPs may adopt a cautious stance that limits their interventions, while other Steering Committee members may hesitate to solicit their input. The results suggest that role clarification should be viewed as an evolving process rather than a one-time step in their integration.

### 5.3. Potential Role Tension for PP Members on the Committee

The presence of PPs on a Steering Committee in complex situations such as budget cuts can lead to tensions around the PP’s role. Are they used by other members of the Steering Committee to validate and endorse difficult decisions, or are they there to raise awareness of the importance of measuring the impact on patients? In this study, no director raised the issue of PPs being exploited in the decision-making. However, it is important to bear in mind the potential for abuse, which could undermine the interest in having PPs participate in Steering Committees. Furthermore, feedback from one of the PPs on how they view their care since participating in the Steering Committee and the concern of one of the directors over ensuring that PPs do not take on an administrative role shows that this is a fine line, and that it is desirable to remain vigilant about the expected role of PPs on the Steering Committee [14,43,44].

### 5.4. Confidentiality and Access to Information: A Structural Tension

Confidentiality emerged as a central and transversal issue. Although the PPs had signed formal confidentiality agreements, their partial access to information, particularly via digital tools, initially limited their ability to participate fully in discussions of strategy. However, these restrictions, often due to external regulatory constraints (e.g., supervisory bodies), were overcome due to the institution’s willingness to move beyond technical issues.

Beyond the functional limitations, this situation could generate mixed messages for PPs: recognized as Steering Committee members, yet excluded from certain sensitive information. This tension underscores the challenges of integrating “hybrid” members into governance structures initially designed for institutional actors. It highlights the need to rethink information-sharing mechanisms in order to reconcile confidentiality requirements with partnership principles, as access to relevant information is essential for active and legitimate patient participation [45].

### 5.5. Effective Participation and Relational Dynamics

The findings show that PPs’ contributions are not systematically solicited during Steering Committee meetings. Their input largely depends on the patient profiles, facilitation dynamics, available time, and relational climate. An important issue is the search for quality in the selection of PPs. If the profile is not right, the experience can be negative for both the committee and the PPs. This can have an impact on other strategic committees within the institution, or even elsewhere in other institutions within the healthcare system, as there is a risk of sharing a bad experience. This finding ties in with the importance of the PPs’ fully understanding their role and not overstepping their remit. Several directors also emphasized the importance of creating formal or informal spaces that encourage PPs to speak up.

In terms of the selection criteria, it is important to have people who are familiar with the institution and who use its services, thereby enabling the sharing of concrete experiences, as well as people who are skilled at interacting with individuals at the strategic level [46]. In addition, supportive moments before and after meetings emerged as key integration levers. These spaces not only clarify the content discussed but also foster the development of mutual trust. However, the study suggests that these practices could be more structured rather than relying solely on individual initiatives.

Furthermore, it is difficult to assess how perceptions of the effects of PPs’ presence on the Steering Committee actually influenced decisions and how this impacted the tactical and operational levels, given the lack of concrete tracking of the decisions made and the effects those decisions had.

### 5.6. Organizational Language as an Implicit Barrier

Frequent use of technical jargon and acronyms represents another obstacle to active PP participation. While some directors acknowledged this issue, it particularly affects PPs, whose ability to navigate the organizational vocabulary conditions the legitimacy of their interventions.

This finding aligns with literature showing that language, especially technical jargon and acronyms, acts as an implicit mechanism of power and exclusion in healthcare organizations, limiting effective participation of actors unfamiliar with these codes [47,48]. The ability to adapt the discourse and clarify the terms used is thus an indicator of organizational maturity in partnership practices.

### 5.7. Symbolic Recognition and Status of Patient Partners

PPs also noted a gap between the discourse affirming their full Steering Committee membership and symbolic elements, such as the absence of their names in official communications or on the institutional website. These symbolic markers, though sometimes perceived as secondary, play a significant role in building a sense of belonging and legitimacy.

This ambiguity reflects a broader tension between being an invited member versus a full member, widely described in literature on participation and governance, and raises questions about institutional recognition of partnerships when deployed at a strategic level [2,3,14].

It is important to remain mindful of the risks of tokenism associated with the presence of PPs on this type of committee [15].

### 5.8. Context of Health System Reform

Finally, the findings show that the reform of the health system and the creation of a new integrated organization (Santé Québec) [49] are not perceived as challenging the principle of PP integration into Steering Committees. In fact, Steering Committees have lost their independence, and even their name, and have become local governance committees. This could reduce institutions’ ability to embrace this innovation unless Santé Québec makes it clear to them that they should do so. Nevertheless, several directors view the partnership as aligned with current network orientations. However, the governance and information security rules associated with this reform constitute additional constraints to be considered.

In this context, evaluating this initiative appears as a potential lever for supporting network-wide reflection and encouraging the deployment of similar mechanisms in other institutions, along with an assessment of the implementation arrangements.

### 5.9. Strengths, Limitations, and Follow Up

This study has several strengths, notably the richness of its qualitative data from cross-perspectives and its analysis of an innovative initiative in a real governance context. Limitations include its focus on a single institution and the relatively recent integration of PPs. This intervention was also implemented during a very complex period. The PPs were introduced during a transformation phase at the institution, but also in the broader context of a reform of the entire healthcare system in Quebec. The Steering Committee therefore had to simultaneously implement a restructuring of their budget and welcome the PPs. Another limitation of this study is that we were unable to include observations from the meetings, document analyses, or quantitative indicators (number of PP interventions per meeting, topics), because we did not obtain authorization to access the documents or attend the meetings as observers. This leads us to conclude that, for future research, it would be desirable to be able to attend Steering Committee meetings, both before and after the implementation of this innovation, and to review the documents resulting from those meetings.

Practically, the results highlight the importance of clarifying and co-constructing roles, formalizing access to information, structuring participation, and symbolically recognizing the PPs’ presence. Addressing these factors appears essential in order to move beyond symbolic participation and achieve a fully operational partnership. Moreover, various improvements have been made since these results were shared with the institution. Notable developments include improved access to documents via Teams, with some restrictions maintained on highly confidential materials; the development of a renewal policy for PPs for three-year terms, a transition period with overlap between outgoing and incoming PPs, and the implementation of peer coaching among the PPs; increased awareness at each Steering Committee meeting of the importance of incorporating PPs’ perspectives into difficult decision-making; and, finally, application of the PP model across all the organization’s Steering Committees.

### 5.10. Reflexivity Statement

MPP, MIN, and CV are researchers who were not involved in the process of bringing the two patient partners onto the Steering Committee, but are researchers working on evaluating partnerships within this institution and in other organizations in Canada and around the world. The other authors (NDW, ABW, MCN, MAC and GD) supported the implementation of this innovation. They were consulted to ensure the proper interpretation of the results but did not interfere with the overall scientific process of conducting the evaluation. In addition, one of the authors (SK) was not involved in any activities related to the partnership at this institution and participated in the data analysis to provide an external, critical perspective. This likely helped minimize research bias, but it is very likely that some bias persists, particularly those related to social desirability.

## 6. Recommendations and Transferability

Based on these results, it is possible to identify contextual success factors that can be mobilized by other institutions interested in introducing such an innovation. These recommendations are structured around 5 steps: (1) Establishing an enabling environment; (2) Selecting and preparing patient partners; (3) Implementing the intervention; (4) Deploying the intervention and continuously improving it; and (5) Sustaining the intervention. Table 4 summarizes the recommendations derived directly from the results and discussion, which can be adopted by institutions interested in implementing such an innovation.

## 7. Future Research

To further strengthen the evidence base and recommendations, additional research should be conducted through mixed-methods case studies combining quantitative (# of PP interventions per meeting, themes pre/post integration using non-participatory observations) and qualitative data (perceptions of the contribution of PPs through document review, interviews and focus groups) before and after the implementation of PPs on Steering Committees, as well as longitudinal studies to examine how the role and influence of PPs on these committees evolve over time. In addition, ethnographic studies would allow us to directly observe Steering Committees over several months in order to analyze: interactions; who speaks up; and the influence on decisions. As for realist evaluations, these would help us better understand which patient partners, in which contexts, influence which decisions, and through what mechanisms.

## 8. Conclusions

The integration of PPs into the Steering Committee at AHM has been positively received and recognized as adding significant value to strategic discussions. PPs bring the patient’s perspective to the Committee’s decision-making, reminding the directors of the real impacts of their choices on patients and reinforcing the hospital’s mission.

However, the study also highlights structural, relational, and procedural tensions limiting the full potential of their participation. Key factors for effective integration include clear definitions of roles and objectives, adequate access to information, the adaptation of the organization’s jargon, and symbolic recognition of the PPs’ status.

These findings underscore that the successful integration of PPs into strategic governance requires more than formal inclusion; it demands ongoing co-construction of roles, continuous support, and organizational readiness to embrace patient expertise. This initiative provides valuable insights for other healthcare institutions seeking to implement patient partnership at a strategic level and helps advance best practices in governance and patient partnership.

## Figures and Tables

**Table 1:** Preparatory and integration phases.

Step	Focus	Period
1. Creating an enabling environment	Conceptualization, leadership endorsement	August 2024–April 2025
2. Selecting and preparing PPs	Recruitment, training, role definition	August 2024–April 2025
3. Implementing the intervention	Onboarding, integration support	April 2024–January 2025

**Table 2:** Steering Committee Directors’ Experience with Patient Partnership and Suggestions to Promote Effective Integration.

Aspect	Answers	No. of Respondents (%)
Positive aspects of the experience	Provides an external perspective from individuals unfamiliar with administrative processes	11 (100%)
	Brings the lived experience of the care pathway	10 (90%)
	Allows the integration of patients’ perspectives and needs into decision-making	9 (82%)
	Adds meaning to the actions taken	8 (73%)
	Helps put proposed solutions into perspective	8 (73%)
Areas for improvement in the experience	Ensure that the perspective provided reflects only the individual’s own experience	5 (45%)
	Ensure that the topics discussed are relevant to patients	4 (36%)
	Define expectations in advance	3 (27%)
Concerns regarding the arrival of PPs	Ability of PPs to take a step back from their own experience	3 (27%)
	Better define the objectives of their presence and expectations of them	2 (18%)
	Should they be present for all discussions?	1 (9%)
Suggestions to promote effective integration	Be aware of best practices in the field	3 (27%)
	Clarify their role, expectations, and frequency of participation	2 (18%)
	Train PPs before they join the Steering Committee	1 (9%)

**Table 3:** Profile of the Patient Partners.

Dimension	PP1	PP2
Hiring date by the establishment	2019	2019
Healthcare experience	Oncology	Surgery
Experience as a Patient Partner	Ongoing treatments	Episodic follow-ups
Professional background	Lawyer	University professor
Number of projects they have participated in at the AHM	17	4

**Table 4:** Key Success Factors for Integrating Patient Partners into Executive Leadership Committees.

Results	Recommendations
**1. Creating an enabling environment**	
Explicit support from senior management	Secure the commitment of the Chief Executive Officer (CEO) and senior leadership.
Provision of dedicated resources to implement the intervention (support from the Department responsible for partnership and from an external organization specialized in partnership)	Obtain support from the department responsible for patient partnership and engagement. Seek support from an external organization with expertise in patient partnership, if needed.
Limited awareness among committee members of the potential contribution of PPs to the executive committee	Assess the expectations and concerns of executive committee members before introducing patient partners (PPs). Provide training on patient partnership principles to Steering Committee members.
**2. Selecting and preparing patient partners**	
Symbolic value of having the CEO sit on the selection committee	Establish a selection committee that includes the CEO, the individual responsible for patient partnership and at least one PP representative.
Complementarity of the two PPs’ profiles	Select PPs based on objective and complementary criteria.
Importance of being able to dialogue with another PP	Recruit at least two PPs.
Recognition of the importance of being trained by PPs (peer-led training)	Provide training in governance and leadership and include a PP as a trainer.
**3. Implementing the intervention**	
Perception of not having had enough preparation time to welcome the PPs	Create opportunities to discuss directors’ expectations and concerns regarding the integration of PPs. Formally introduce PPs to executive committee members.
Difficulty understanding the vocabulary and acronyms, as well as accessing documents, leading to difficulties in participating in discussions	Provide a list of acronyms and definitions to PPs and directors.
PPs’ recognition of the importance of being supported, particularly at the outset	Appoint the director in charge of partnership as a mentor for the PPs.
**4. Deploying the intervention and continuously improving it**	
Recognition of the QD’s (Quality Director’s) watchdog role in ensuring the deployment proceeds under good conditions	Appoint a member of the executive committee responsible for ensuring that the integration process proceeds under favorable conditions.
Difficulty accessing documents	Establish a formal status and role description for PPs. Grant PPs the necessary technological and organizational access to participate fully in committee activities.
PPs’ frustration regarding their status on the committee, marked by discrepancy between the discourse of being integrated into the Steering Committee and situations demonstrating otherwise	Officially recognize the role and mandate of PPs on the Steering Committee in all official governance documents (e.g., listing their names on the organization’s website, including them in meeting minutes and official documents). Avoid communication channels that exclude PPs from discussions and decision-making processes.
No dedicated time allocated for PPs	Ensure that PPs are given dedicated opportunities to contribute during each executive committee meeting.
Lack of recognition of their contribution to the Steering Committee	Create regular opportunities for direct dialogue between the CEO and PPs to discuss their experience and identify areas for improvement.
**5. Sustaining the intervention**	
Contribution of the evaluation conducted to improving processes	Conduct ongoing evaluations of the contributions of PPs.
Lack of evaluation of the effects of their contribution on decisions at other levels of governance and at the clinical level	Implement mechanisms to track how discussions involving PPs influence Steering Committee decisions. Evaluate the impact of PPs not only at the Steering Committee level but also across departments and at tactical and operational management levels.

## Data Availability

Data available on request due to restrictions.

## Supplementary Materials

The following supporting information can be downloaded at: https://www.mdpi.com/article/10.3390/healthcare14132021/s1. Supplementary File S1: Interview guide; Supplementary File S2: Survey of Steering Committee members in preparation for the integration of patient partners; Supplementary File S3: selection criteria used to recruit the PPs.

## Abbreviations

AHM: Academic hospital in the heart of Montréal; CEPPP: Centre of Excellence on Partnership with Patients and the Public; DQEPE: Direction de la qualité, de l’évaluation, de la performance et de l’éthique (Quality, Evaluation, Performance and Ethics Department); PP: patient partner; QD: Quality Department; SRQR: Standards for Reporting Qualitative Research; TCPS2: Tri-Council Policy Statement; WHO: World Health Organization; OECD: Organisation for Economic Co-operation and Development.
