# Supplementary material for: Evaluation of the Implementation and Contribution of Patient Partners on a Steering Committee at a University Hospital in the Province of Québec, Canada
Source: Healthcare (Basel). 2026 Jul 7;14(13):2021. doi: 10.3390/healthcare14132021 (PMC13360805; doi:10.3390/healthcare14132021)
Supplement: Supplementary file 1 [file healthcare-14-02021-s001.zip › file s1_Interview Guide.pdf]

Interview Guide

Patient partner or caregiver candidate for a one-year mandate on the CHUM Steering Committee

Quality, Evaluation, Performance and Ethics Directorate

Centre hospitalier de
l'Université de Montréal

January 31, 2024

CHUM
Centre hospitalier
de l'Université de Montréal

INTERVIEW GUIDE

PATIENT PARTNER (OR CAREGIVER) - PP

PATIENT PARTNERSHIP OFFICE, HEALTH PROMOTION SERVICE

QUALITY, EVALUATION, PERFORMANCE AND
ETHICS DIRECTORATE (DQEPE)

PP CANDIDATE NAME:

DATE/TIME:

SELECTED: YES NO

Date of availability:

Specific expectations or needs to consider according to the selected candidate's situation:

___

___

___

___

INTEREST AND MOTIVATION 1

1. What motivates you to submit your application to sit specifically on the CHUM Steering Committee?

   - Role associated with the position?
   - New responsibilities – new challenge?
   - Topics of concern to the candidate?

____

____

____

____

____

____

____

____

2. What is your understanding of the mandate of the CHUM Steering Committee?

____

____

____

____

____

____

____

____

3. What are your expectations of the Steering Committee? What would you need in order to fully play your role as a patient partner?

____
____
____
____
____
____
____
____
____
____

BEHAVIOUR IN POSITIVE AND COMPLEX SITUATIONS

4. Could you describe the best experience you have had as a patient partner?

____
____
____
____
____
____
____
____

5. Could you describe the most difficult or most negative experience you have had as a patient partner, and how you managed that situation?

6. If your application is selected, are there any particular or specific needs related to your situation that you would like to let us know about to facilitate your participation on the Steering Committee?

7. Do you have any questions or comments?

ASSESSMENT OF COMPETENCIES AND AVAILABILITY

Personal abilities/attitudes to assess:

- Communicates clearly?
  Yes ☐ No ☐
- Has interpersonal skills that facilitate collaboration (listening, empathy, etc.)?
  Yes ☐ No ☐
- Demonstrates a desire to help others and contribute to an objective that goes beyond their own health condition?
  Yes ☐ No ☐
- Has experience that supports an understanding of cross-cutting organizational issues.
  Yes ☐ No ☐
- Demonstrates good resilience with respect to their own health care situation in order to share their experiences?
  Yes ☐ No ☐
- Demonstrates a desire to be involved with other patients, work teams, and governance?
  Yes ☐ No ☐

Availability and capacity to participate

- Available on Tuesday mornings? Yes ☐ No ☐
- Able to participate: In person ☐ Virtually ☐

INTERVIEW COMMENTS

REFERENCES

CEPPP, 2020, online: https://ceppp.ca/wp-content/uploads/2021/01/USSQ_Guide-pratique-SPPP-en-recherche_V1.pdf

APPENDIX

Backup questions

GENERAL INTRODUCTION AND PROFESSIONAL EXPERIENCE

8. Could you give us a brief general introduction to yourself and briefly describe your academic background and professional career path?

- Consider emphasizing elements related to management and the understanding of cross-cutting organizational issues.

EXPERIENCE AS A PATIENT (OR CAREGIVER) IN CARE/SERVICES AND AS A PATIENT PARTNER

9. What led you to become a patient partner? Tell us about your involvement at the CHUM and how it led you to become a partner in your own care.

____

____

____

____

____

____

____

____

10. In your view, what is the role of patient partners within the health and social services system? And within the Steering Committee?

____

____

____

____

____

____

____

____

11. As a patient partner, have you ever been in a situation of conflict with your health professional(s) or other stakeholders? How did you manage these situations?

____
____
____
____
____
____
____
____

12. How comfortable would you feel sitting around a table with directors while holding the title of “patient or caregiver”? On a scale of 1 to 10, how comfortable would you be sharing your perspective as a patient on the same footing as the Committee directors? (Assess the risk of feeling intimidated)

____
____
____
____
____
____
____
____
