# Supplementary material for: Evaluation of the Implementation and Contribution of Patient Partners on a Steering Committee at a University Hospital in the Province of Québec, Canada
Source: Healthcare (Basel). 2026 Jul 7;14(13):2021. doi: 10.3390/healthcare14132021 (PMC13360805; doi:10.3390/healthcare14132021)
Supplement: Supplementary file 1 [file healthcare-14-02021-s001.zip › file s2_Survey of Steering Committee members in preparation for the integration of patient partners.pdf]

Survey in preparation for the integration of patient partners into the Steering Committee

Survey of Steering Committee members in preparation for the integration of patient partners

In preparation for the integration of two patient partners into the Steering Committee, a joint presentation on the patient partnership approach will be offered by the Centre of Excellence on Partnership with Patients and the Public and the Patient Partnership Office of the DQEPE.

We invite you to complete this 5-minute survey. The data will be treated confidentially and used solely to adapt the presentation to the Committee's needs.

Thank you for your valuable collaboration!

* Required

* This form will record your name; please enter your name.

1. Have you ever collaborated with a patient partner as part of a project or committee? *

Yes

No

2. If yes: Could you share the positive aspects of this experience?

3. If yes: Could you share the areas for improvement from this experience?

4. Do you have any questions or topics you would like to bring to our attention in preparation for this partnership experience on the Steering Committee?

5. Would you like to share any needs with us so that they can be taken into account when preparing the presentation on patient partnership?

6. Do you have any other comments to submit?
