# Supplementary material for: Evaluation of the Implementation and Contribution of Patient Partners on a Steering Committee at a University Hospital in the Province of Québec, Canada
Source: Healthcare (Basel). 2026 Jul 7;14(13):2021. doi: 10.3390/healthcare14132021 (PMC13360805; doi:10.3390/healthcare14132021)
Supplement: Supplementary file 1 [file healthcare-14-02021-s001.zip › file s3 selection criteria used to recruit the PPs.pdf]

file s3: Patient Partner Selection Criteria

Criterion	Description
Clinical profile	Having had one or more care episodes at the CHUM; significant experience as a patient partner or caregiver partner in cross-cutting organizational projects; or having served on a management committee of a health and social services institution.
Profile representative of the diversity of CHUM's patient population	Reflects, where possible, diversity in terms of age, ethnic background, gender, condition, pathology, etc.
Interests of the patient partner or caregiver	Interest in CHUM's mission and vision, healthcare system issues, patient satisfaction, and quality and safety of care.
Experience as a patient partner (PP) in projects and management committees	Candidates are assessed based on their experience, knowledge, competencies, understanding of cross-cutting issues, and contribution to problem-solving.
Professional background	Assets include experience in human resources, financial or material resource management, project management, etc.
Availability	Must be available every Wednesday morning from 10:00 a.m. to 11:30 a.m.
Participation format	Must be flexible and able to participate in a hybrid format according to the Steering Committee's needs (in person or virtually).
Required equipment	A personal computer with audio and a camera.
Benefits	Access to free snacks and meals at the cafeteria, a free parking permit, and a CHUM email address to follow the Steering Committee's work.
